# Long-Term Experience with Radiofrequency-Induced Hyperthermia Combined with Intravesical Chemotherapy for Non-Muscle Invasive Bladder Cancer

**DOI:** 10.3390/cancers13030377

**Published:** 2021-01-20

**Authors:** Iris S. G. Brummelhuis, Yvonne Wimper, Hilde G. J. M. Witjes-van Os, Tom J. H. Arends, Antoine G. van der Heijden, J. Alfred Witjes

**Affiliations:** 1 Department of Urology, Radboud University Medical Center, 6500 HB Nijmegen, The Netherlands; yvonne.wimper@radboudumc.nl (Y.W.); hilde.witjes-vanos@radboudumc.nl (H.G.J.M.W.-v.O.); toine.vanderheijden@radboudumc.nl (A.G.v.d.H.); fred.witjes@radboudumc.nl (J.A.W.); 2 Department of Urology, Meander Medical Centre Amersfoort, 3813 TZ Amersfoort, The Netherlands; tjh.arends@meandermc.nl

**Keywords:** bladder cancer, NMIBC, hyperthermia, chemohyperthermia, chemosensitization, RITE, mitomycin-C, epirubicin, Synergo

## Abstract

**Simple Summary:**

Non-muscle invasive bladder cancer is a disease that frequently recurs, despite standard bladder instillations with chemotherapeutic agents or immunotherapy. When the disease recurs despite treatment with standard bladder instillations, urology guidelines recommend surgical removal of the bladder. This major operation often comes with complications or even death. Therefore, patients are often unfit or unwilling to undergo this operation. In this study, we present the treatment outcome of “radiofrequency-induced hyperthermia combined with intravesical chemotherapy”, which are bladder instillations with a chemotherapeutic agent, while simultaneously heating the bladder wall with microwave radiation to fever temperature. We compare the outcomes of two tumor subtypes of non-muscle invasive bladder cancer and two doses of chemotherapeutic agent. We conclude that this therapy is effective and safe in both types of non-muscle invasive bladder cancer patients in whom standard bladder treatments have failed. The high dose should be used if patients have a tumor at therapy onset.

**Abstract:**

Background: The recurrence rate of non-muscle invasive bladder cancer (NMIBC) is high, despite intravesical treatments. Importantly, patients are frequently unfit or unwilling to undergo a recommended radical cystectomy when standard intravesical treatments fail, due to the substantial risk of morbidity and mortality. For these patients, radiofrequency-induced hyperthermia combined with intravesical chemotherapy (RF-CHT) has shown promising results. We aim to determine treatment outcomes and assess the effect of (ablative) dose. Methods: 299 intensively pretreated patients treated with RF-CHT were included in safety analysis. Of these, 274 patients who fulfilled induction treatments were included in efficacy analysis. Six-month complete response (CR) and durable response were reported for (concomitant) carcinoma in situ (CIS) patients and recurrence-free survival (RFS) for papillary patients. Results: For CIS, six-month CR-rate was 56.0%; and durable response rates were 79.7%, 66.5%, and 40.3% at one-, two- and five-year, respectively. RFS rates for papillary patients were 77.9%, 57.5%, and 37.2%, respectively. Patients treated with ablative dose are less likely to develop recurrence (adjusted Hazard Ratio 0.54, *p* = 0.01), compared to adjuvant dose. Conclusions: RF-CHT is effective in NMIBC patients in whom standard intravesical treatments have failed and should be considered in patients who are unwilling or unfit to undergo radical cystectomy. Patients with CIS or residual papillary tumor at baseline benefit from ablative dose.

## 1. Introduction

Non-muscle invasive bladder cancer (NMIBC) is a significant health problem in the European Union (EU). With an estimated 152,000 new cases in 2018, bladder cancer is the fifth most common cancer in the EU [1]. Besides that, bladder cancer is the most expensive malignancy to treat per capita, costing the EU nearly €5 billion per year [2,3]. The largest part of bladder cancer patients, about 75%, present with NMIBC, including papillary tumors (Ta), carcinoma in situ (CIS or Tis) and tumors invading the subepithelial connective tissue (T1) [4]. 

The standard treatment for NMIBC has significant downsides. According to the guidelines, NMIBC should be treated with transurethral resection of the bladder tumor (TURB) followed by an immediate single postoperative instillation of chemotherapy [4]. Adjuvant intravesical instillations with Bacillus Calmette-Guérin (BCG) or chemotherapy are required depending on the risk group. However, worldwide BCG shortage has evolved over the last decade [5]. More importantly, despite intravesical treatment, intermediate and high risk NMIBC patients have a recurrence and progression probability up to 52% and 20% (high risk) at five years, respectively [6]. When intravesical treatment in high-risk patients fail, the guidelines recommend a radical cystectomy with urinary diversion [4]. 

The key problems of this complex surgery are the 90-day mortality rates of 2.3–9% and perioperative morbidity rates of up to 60% [7,8,9]. Late complications, such as a loss of renal function, are also frequently observed [10]. Moreover, the physical and social quality of the life of bladder cancer patients is significantly diminished after radical cystectomy [11]. Despite the fact that bladder cancer is more prevalent in the older population—most bladder cancer patients are over 70 years of age—older patients are less likely to undergo radical cystectomy [12]. The reasons for impaired utilization of radical cystectomy are frailty, i.e., decline in physical fitness and comorbidities, as well as concerns about burdens from surgery including functional recovery and stomal care. Therefore, there is a clear need for alternative, bladder-sparing treatments for NMIBC patients who are unfit or unwilling to undergo a radical cystectomy. 

Microwave-induced chemohyperthermia (RF-CHT) is a device-assisted technique applied to improve efficacy of intravesical chemotherapy and has shown encouraging results in treatment of NMIBC. This treatment includes circulation of mitomycin-C (MMC) or epirubicin in the bladder and simultaneous hyperthermia (HT) of the bladder wall at 40.5–44 °C using radiofrequency (RF) [13]. HT is known to contribute to direct and indirect DNA damage [14], to trigger an anti-cancer immune response [15], to increase drug concentration in bladder cancer tissue [16] and to potentiate the effect of chemotherapeutic agents on bladder cancer cells [17]. 

Following the current European Association of Urology (EAU) guideline, RF-CHT can be considered in NMIBC patients in whom previous intravesical treatment failed and who are unable or unwilling to undergo radical cystectomy [4] but ablative (high) dose for patients with tumor at baseline is not standard and optimal treatment duration is unknown. A recent randomized controlled trial (RCT) in BCG-unresponsive NMIBC showed a non-significant higher two-year disease-free survival (DFS) in papillary patients who received RF-CHT compared to patients treated with the institutional standard (including BCG, MMC alone, or with electromotive drug administration; 53% vs 24%, *p* = 0.11) [18]. This is in line with a previous RCT comparing RF-CHT and BCG (in BCG naïve patients) [19]. In contrast, DFS was significantly lower in CIS patients treated with RF-CHT than those treated with the institutional standard. However, only half the MMC dose (i.e., adjuvant dose) typically prescribed for CIS patients (ablative dose) was used [20,21,22,23]. 

The aim of this study was to determine treatment outcome including complete response (CR) and durable response (both for CIS), recurrence free survival (RFS; for papillary patients), overall survival (OS), relative survival (RS), and cancer specific survival (CSS) for the largest cohort treated with RF-CHT so far. Additional objectives were the assessment of the effect of chemotherapeutic dose on treatment outcome, the optimal treatment duration and evaluation of safety and tolerability. Lastly, the treatment outcome in the BCG refractory subgroup was assessed. 

## 2. Results

### 2.1. Patients

All 299 intermediate and high risk NMIBC patients who received RF-CHT using intravesical MMC or epirubicin were included for tolerability and safety analysis. Twenty-five patients received less than six treatments due to the following reasons: six patients started RF-CHT treatment less than six weeks before this study was conducted, one patient appeared to be metastasized at start of RF-CHT treatment, one patient stopped RF-CHT due to complaints caused by residual bladder tumor, in three patients RF-CHT catheter placement was not feasible due to urethral stricture, and 14 patients stopped because of local side-effects (see tolerability and safety section). Thus, 274 received at least six treatment sessions and were eligible for the efficacy analysis. 

**Figure 1:**
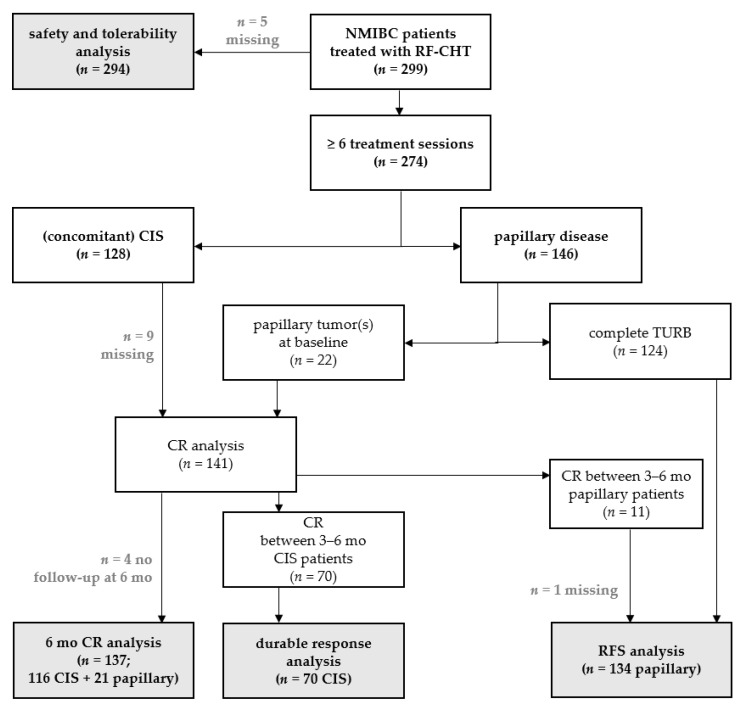
Flow-chart for this retrospective study including all non-muscle invasive bladder cancer (NMIBC) patients treated with RF-CHT.

The patient and tumor characteristics are summarized in Table 1. The median follow-up was at 55.5 months. At baseline, 128 patients (46.7%) had CIS with or without concomitant papillary tumor and 146 had papillary tumor (Ta/T1) only. All patients with low grade (LG) Ta tumors had highly recurrent disease. In most patients, papillary tumors were resected before start of RF-CHT, except for 22 (without concomitant CIS). These 22 papillary patients all received an ablative dose (40 mg MMC or 50 mg epirubicin twice per treatment session) and were included in the CR analysis (Figure 1). Of all patients with CIS at baseline, 80 patients (62.5%) received an ablative dose (40 mg MMC or 50 mg epirubicin twice per treatment session) and 48 patients (37.5%) an adjuvant dose (20 mg MMC or 30 mg epirubicin twice per treatment session). 

The majority of patients included in the efficacy analysis have been treated previously with intravesical instillations (85.4% BCG, 50.4% MMC) and for 12 patients (4.4%) RF-CHT was the initial treatment. From the 234 patients who previously received BCG, 178 (65.0%) had BCG refractory disease and 21 (7.7%) were BCG intolerant (i.e., discontinued BCG due to serious side effects). For five patients it was not documented whether they were treated with BCG previously, and in 35 patients who were treated with BCG the reason for BCG discontinuation was not documented.

### 2.2. Efficacy 

#### 2.2.1. Complete Response

The CR rate at six months was 56.0% for CIS patients and 52.4% for patients with residual papillary tumor at baseline (Table 2). Ablative chemotherapeutic dose was associated with higher six-month CR rate, compared to adjuvant dose, however, not significant (adjusted odds ratio (OR) 0.49, *p* = 0.08). (Concomitant) CIS at baseline also improved the six-month CR rate (adjusted OR 0.35, *p* = 0.10). 

Follow-up data regarding CR was missing for nine patients because of the following reasons: six patients started RF-CHT less than three months before inclusion in this study (thus before first follow-up), one patient died before first follow-up because of an urosepsis (not related to RF-CHT treatment) and two patients were lost to follow up (reason unknown). Specifically, data regarding pathology or cystoscopy and cytology at six months were missing for four patients without a documented reason. From the 137 patients included in the CR analysis, 81 (70 CIS and 11 papillary) patients had a CR at follow-up at three or six months and were included in the durable response (and in case of papillary disease in the RFS) analysis. 

The CR rate at six months in the BCG refractory subgroup was 54.5% for CIS patients (48/88 with available follow-up data at six months) and 43.8% (7/16) for patients with residual papillary tumor at baseline.

#### 2.2.2. Durable Response and Recurrence-Free Survival

Durable response rates of patients with (concomitant) CIS at one-, two-, and five-year were 79.7%, 66.5%, and 40.3%, respectively (Table 3). The RFS rates for patients with papillary disease at these intervals were 77.9%, 57.5%, and 37.2%, respectively. 

**Figure 2:**
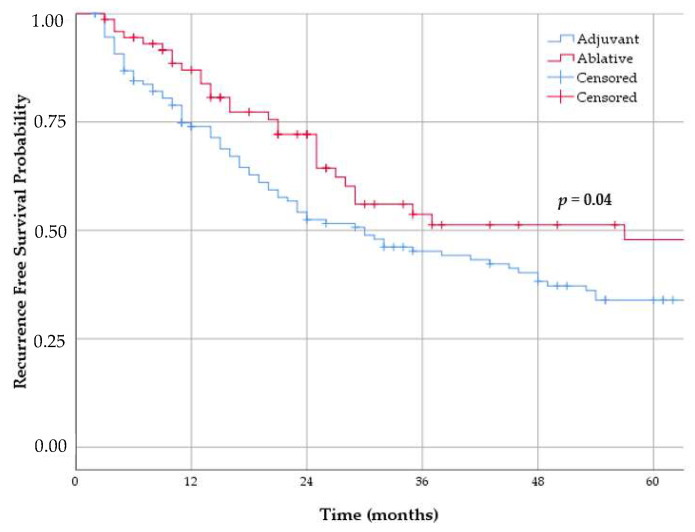
Recurrence free survival (RFS) and durable response combined after RF-CHT for ablative dose group and adjuvant dose group. Patients treated with ablative dose had significant better RFS and durable response (univariable analysis *p* = 0.04).

Univariable analysis demonstrated that ablative dose significantly improved RFS and durable response rate, compared to adjuvant dose (*p* = 0.04, Table 3 and Figure 2). Also, after adjusting for possible confounders (see method section), patients treated with ablative dose showed to be significantly less likely to develop a recurrence (adjusted hazard ratio (HR) 0.54, *p* = 0.01, Table 4). CIS patients have, from the moment of CR on, a similar probability of developing recurrence as papillary patients (adjusted HR 0.94, *p* = 0.81). Prior BCG treatment increased the risk of recurrence (adjusted HR 2.07, *p* = 0.04). 

In the RFS analysis, one patient was lost to follow up because RF-CHT treatment was discontinued due to side effects and the patient was referred for further follow-up. 

In the subgroup of BCG refractory patients, durable response rates (for CIS patients, *n* = 52) were 79.2%, 65.5%, 38.7%, and RFS rates (for papillary patients, *n* = 68) were 72.5%, 54.0%, 31.7%, at one-, two- and five years, respectively. 

#### 2.2.3. Progression

In total, 22 (8.5%) of all patients progressed to MIBC, of whom 20 had a high-grade tumor prior to RF-CHT and all 22 patients previously have been treated with BCG (21 BCG refractory, 1 unknown reason for BCG discontinuation). Eleven (4.3%) patients had distant metastases up to one year after treatment. 

#### 2.2.4. Overall Survival, Relative Survival and Cancer Specific Survival

OS, RS, and CSS rates at five and ten years of patients included in the efficacy analysis are shown in Table 5. Within the BCG refractory subgroup, the OS rates were 70.5% and 43.9%, RS rates 78.6% and 57.5% and CSS rates 85.7% and 73.1%, at 5 and 10 years, respectively. More specific data for the 274 patients included in the efficacy analysis is described in Appendix A.

#### 2.2.5. Treatment after RF-CHT

During the mean follow-up period of 55.5 months, 80 patients (29.2%) received a radical cystectomy with or without neoadjuvant chemotherapy (15 patients (5.5%) revealed to have progression to muscle invasive disease); 5 patients (1.8%) received systemic chemotherapy only; 4 patients (1.5%) received chemoradiation, with or without a TURB; and 12 patients (4.4%) received other intravesical therapy after RF-CHT. The bladder preservation rate for this follow-up period was thus 70.8%. The median time from last TURB to cystectomy was 18 months. In 76.0% of patients, a radical cystectomy could be prevented for two years from last TURB, and in 61.1% a radical cystectomy could be prevented for five years. OS rate of patients who received a radical cystectomy was 71.0% at five years and 42.6% at ten years.

#### 2.2.6. Outcome after “Completed” RF-CHT Treatment

In 44 patients, no significant recurrence occurred during treatment which was “completed” after a median treatment period of 30.5 months (range from 1–7 years). Fifteen patients developed a recurrence after completed treatment. Out of 24 patients who received treatment less than two years, 12 developed a recurrence, whereas only 3 out of 20 patients who had been treated for more than two years developed recurrence after completed treatment (*p* = 0.02).

### 2.3. Tolerability and Safety 

Of all the treated patients, 94.2% experienced at least one adverse event. During treatment, spasms and pain were observed in 62.2% and 27.8% of the patients, respectively (Table 6). Dysuria and hematuria were observed in 53.1% and 29.9% following treatment, respectively. From all adverse events, the highest grade ever reported per patient was mild (Common Terminology Criteria for Adverse Events (CTCAE) grade 1) in 62.6% and moderate (CTCAE grade 2) in 32.9%. In total, 30 (10.2%) patients experienced once a severe (CTCAE grade 3) adverse event. Thirty-four patients (11.4%) discontinued treatment due to side effects. Anticholinergics were used around the treatment sessions by 20.5% of the patients. Significantly less patients who received an ablative dose reported pain or dysuria, compared to adjuvant dose (19.5% vs. 34.8%, *p* < 0.01; and 42.1% vs. 62.1%, *p* < 0.01). Incontinence was more frequently observed in patients who received an ablative dose, compared to adjuvant dose (10.5% vs 2.5%, *p* < 0.01). Six (2.0%) patients reported moderate incontinence for which medication was indicated. The number of patients with spasms, nocturia, and hematuria did not differ significantly between treatment doses. In total, 18.7% received epirubicin, which was in 15.4% of all patients because of an allergy to MMC. Data of five patients (of 299) regarding adverse events were missing.

## 3. Discussion

We report the treatment outcomes of an intermediate- and high-risk NMIBC patients cohort treated with RF-CHT. This is the largest cohort treated with RF-CHT ever reported, with a median follow up of 55.5 months. The six-month CR rate of patients with (concomitant) CIS at baseline was 56.0%; and durable response rate at two years was 66.5%. One-, two- and five-year RFS for papillary patients were 77.9%, 57.5% and 37.2%, respectively. Patients treated with ablative dose are less likely to develop recurrence (adjusted HR 0.54, *p* = 0.01) and ablative dose showed a trend towards better CR at six months significant (adjusted OR 0.49, *p* = 0.08). Patients treated with RF-CHT for two years or more had less recurrences after treatment completion, than those treated for less than two years. 

The presented treatment outcomes are fair, when compared to the generally accepted efficacy of (new) bladder-sparing treatments for patients in whom previous intravesical treatment has failed. The majority of patients included in this study had several poor risk factors such as previous treatment with BCG (thus BCG refractory or intolerant), high grade NMIBC, and/or high tumor recurrence frequency (≥1 per year) [6,23] Moreover, 46.7% of the patients had (concomitant) CIS. The International Bladder Cancer Group recommends that a CR and RFS rates of at least 50% at 6 months, 30% at 12 months, and 25% at 18 months are clinically meaningful in single-arm study designs for BCG refractory NMIBC [24]. 

Putting our results into perspective, pembrolizumab, a systemically administered PD-1 antagonist studied in BCG-unresponsive CIS and approved for this indication by the Food and Drug Administration (FDA) had a CR rate at three months of 40.6% (39 patients from 96 included in efficacy analysis) [25,26]. Among patients with CR, 18.8% (18 out of 96) had a duration of response of 12 months or more. Another strategy that has been studied in phase II and III trials is intravesical instillation of nadofaregene firadenovec, an adenoviral vector with the human IFN-α2b gene and Syn3 which incorporates into the cellular DNA and induces synthesis and expression of the IFN-α2b protein [27]. The phase III results revealed a three-month CR of 53.4% (55 out of 103) in CIS patients, of whom 25 maintained CR at 12 months (24.3%). One-year RFS for papillary patients was 43.8%. A third therapy that received attention recently, is sequential intravesical gemcitabine and docetaxel as rescue therapy in recurrent NMIBC patients with a history of BCG treatment, presented by Steinberg et al. [28]. A retrospective multi-institution analysis including 276 patients demonstrated a one- and two-year RFS 60% and 43% for patients with (concomitant) CIS, while one- and two-year RFS for patients with papillary disease was 62% and 51%, respectively. Additionally, in the subgroup with high-grade BCG-refractory NMIBC, CIS patients and patients with papillary disease had a two-year RFS of 50% and 58%, respectively. Our CR and RFS results seem similar to the outcomes of these studies.

The HYMN study is a recent RCT published by Tan et al., which assessed the efficacy of RF-CHT (combination with MMC) in patients previously treated with BCG, compared to the institutional standard (a second cycle of BCG, MMC alone or MMC combined with electromotive drug administration) [18]. Primary outcome measures were DFS (for CIS and papillary patients) and CR at three months (for CIS patients). “DFS was determined as time from randomization to earliest detection of histologically confirmed recurrence, positive urinary cytology, or death.” Their study showed a non-significant higher two-year DFS in papillary patients who received RF-induced HT and MMC. In contrast, DFS was significantly lower in CIS patients treated with RF-induced HT and MMC than those treated with the institutional standard. CR at three months was 30%. However, both the International Bladder Cancer Group and the FDA recommend using RFS as primary end point for papillary tumors and CR at six months for (concomitant) CIS patients, not DFS [24,29]. After CR, durable response may be determined for CIS patients. Both RFS and CR were not significantly different in RF-CHT compared to the institutional standard. Moreover, the HYMN study used adjuvant dose of MMC (twice 20 mg) for CIS patients instead of an ablative dose (twice 40 mg), which could affect treatment outcome. 

We had the opportunity to compare the effect of adjuvant and ablative dose on treatment outcome, including patients with residual tumor (mainly CIS) at baseline. The dose for these patients was increased as soon as it became clear that these patients benefited from a higher dose [20], a few years after we started with RF-CHT treatments. This was in line with other studies investigating the effect of HT combined with intravesical chemotherapy in NMIBC patients [21,22,23]. Furthermore, some patients with CIS received an adjuvant dose due to side effects caused by the chemotherapeutic agent. An ablative dose showed a trend towards improved CR and significantly increased RFS and durable response, a logical result since a higher dose should have more effect than a lower dose. A recent retrospective study also reported a trend towards improved CR in CIS patients treated with an ablative dose RF-CHT (*p* = 0.06) [23]. These data highlight the importance of an ablative dose in patients with residual tumor (CIS or papillary) at baseline. RCTs adopting an ablative dose for these patients and assessing appropriate end points (CR for CIS and RFS for papillary tumors) are urgently needed.

(Concomitant) CIS patients have, from the moment of CR on, a similar probability of developing recurrence as papillary patients (adjusted HR 0.94, *p* = 0.81). In contrast, the six-month CR rate is higher in patients with CIS at baseline (adjusted OR 0.35, *p* = 0.10). However, it should be mentioned that only 21 papillary patients (with unresected tumor) were included in the CR analysis, which makes is difficult to draw hard conclusions. 

Progression, OS, RS, and CSS reported in our high-risk population, far most BCG refractory, are in line with other series. Importantly, we report the progression to MIBC in only 8.5% during a median follow-up of 55.5 months. High grade, papillary patients (without CIS), who are subjected to BCG treatment for the first time, have a probability of progression at five year of 4.6–19.8% [6]. The progression rate in the retrospective analysis of Steinberg et al. was 7.6% (21/276) [28]. The five-year OS of all 274 patients included in the efficacy analysis was 72%, and the five-year OS of the 80 patients who received a radical cystectomy in our cohort was 71%. In comparison, five-year OS was 74% for all NMIBC patients who received radical cystectomy in the Netherlands from 2011 to 2016 (100–120 patients per year: data from the Dutch Cancer Registry). A study including NMIBC patients who received a radical cystectomy due to a high likelihood of progressing reported a five-year OS rate of 63% [30]. RS rate of our patient cohort at five years was with 81% similar to the RS of 80% for all NMIBC patients who received radical cystectomy in the Netherlands from 2011 to 2016. Lastly, five-year CSS in our cohort was 86.6; corresponding to series that reported five-year CSS of 90% in BCG refractory NMIBC patients that underwent radical cystectomy [31]. Despite the fact that we did not compare RF-CHT to radical cystectomy in this one-arm study, survival rates appear to be comparable to radical cystectomy.

The concern remains that RF-CHT eventually may delay an unavoidable radical cystectomy, and possibly complicate future surgery and oncological outcome. In our series 80 patients (29.2%) underwent radical cystectomy after a mean follow up of 55.5 months. A recent study published by Sri et al. focused on operative challenges and oncological outcome in high risk, BCG refractory NMIBC patients undergoing radical cystectomy after RF-CHT, compared to a comparable group of patients who received radical cystectomy immediately after BCG failure [32]. After a mean follow up of 24 months, 36 out of 138 patients (26%) underwent radical cystectomy. No significant differences were found in duration of operation, intraoperative blood loss, length of hospital stay or 90-day readmission rate between the two groups; all surrogate markers to predict intraoperative difficulty. Moreover, no significant differences in time to recurrence, OS and CSS were observed. The authors conclude that RF-CHT does not result in a technically more challenging radical cystectomy and does not compromise oncological outcome compared to patients who undergo immediate cystectomy.

Adverse events due to RF-CHT were common, with the majority being mild to moderate and limited to the lower urinary tract. From all patients, 94.2% experienced an adverse event at least once. The maximum CTCAE grade reported per adverse event was recorded for each patient. The highest grade adverse event reported was severe in 10.2%, moderate in 32.9%, and mild in the remaining 62.6% of the patients. No life-threatening adverse events or deaths related to RF-CHT were reported. However, 9.4% of patients discontinued treatment due to side effects. This rate is comparable to that of patients who do not tolerate BCG treatment: eight percent of patients treated with BCG discontinued therapy due to toxicity [33]. Where most adverse events from RF-CHT are limited to the lower urinary tract, 30.6% of BCG-treated patients also report systemic adverse events (in addition to local side effects in 62.8% of patients). Significantly more patients who received an ablative dose reported incontinence, which was typically transient. Only six patients reported moderate incontinence for which medication was indicated, the other 12 patients reported mild incontinence. Unexpectedly, significantly more patients treated with the adjuvant (lower) dose reported pain and dysuria. Since our patient cohort was intensively pretreated with multiple TURBs and intravesical instillations, one explanation for this observation could be that urologists more frequently prescribed an adjuvant dose (with presumed fewer side effects) for patients with pre-existing lower urinary tract symptoms. Moreover, we suspect that the temperature and duration of treatment greatly impacts these numbers, but their role has not been assessed. Allergy to MMC was reported in 15.4% of patients, compared to 9% of patients treated with cold MMC instillations in literature [34]. Anticholinergics were used around the treatment sessions in 20.5% of the patients. Since most adverse events are limited to the lower urinary tract, anticholinergics may be considered in each patient. 

Since there is still no consensus about the optimal RF-CHT treatment schedule, there was a high variation in maintenance scheme and treatment duration during 18 years of our increasing experience. Patients who received treatment for more than two years, had significantly less recurrences after completed treatment than patients treated for less than two years (*p* = 0.02). However, patients that received treatment for at least two years without recurrence may have a better prognosis than patients who had treatment for less than two years. Nevertheless, we recommend treatment with RF-CHT for at least two years. 

The strengths of this study are that we report 18 years’ experience with RF-CHT applied in the largest cohort patients treated with this treatment published to date. Importantly, in this manuscript we provide a reasonable alternative treatment for frail NMIBC patients who are unfit or unwilling to undergo radical cystectomy. Moreover, the effect of ablative and adjuvant dose on treatment outcome could be assessed and shows that the use of an ablative dose in patients with CIS or residual papillary tumor is essential. 

The limitations of this study include those inherent to the retrospective study design, including risk of selection bias, information bias, and underreporting of side effects. There were missing values regarding cystoscopy and cytology. Moreover, for some patients the chemotherapeutic dose was changed during treatment, for which we did not correct. 

## 4. Materials and Methods 

### 4.1. Dataset Characteristics 

Out of 324 patients that received RF-CHT between November 2001 and January 2020, we reviewed all available 299 medical records. Paper files of 25 patients were missing. The inclusion criteria for the study population for efficacy analysis were patients with histologically proven NMIBC who received at least six RF-CHT instillations at Radboud University Medical Center. For evaluation of tolerability and safety analysis, all 299 treated patients were included. 

### 4.2. Treatment Schedule and Follow-Up 

Patients received six-weekly induction treatment sessions with RF-CHT, followed by a maintenance regimen of one instillation every six weeks for the first year in case of good response. The treatment interval was extended depending on the urologist expertise. In general, patients received one instillation every 8 weeks for the second year and once in the 12 weeks thereafter. RF-induced HT was conducted using the Synergo SB-TS 101 System (Medical Enterprises Europe B.V., Amstelveen, The Netherlands). A treatment session included two 30-min cycles with intravesical MMC (20 or 40 mg) or epirubicin (30 or 50 mg) at 40.5–44 °C. Typically, papillary tumors were resected prior to RF-CHT. Chemotherapeutic dose changed over time: in the beginning all patients received an adjuvant dose (20 mg MMC of 30 mg epirubicin) twice per treatment session. Later it became clear that for tumor ablation a higher dose was required. In the case of MMC allergy, patients switched to epirubicin. Follow-up included cystoscopy and urinary cytology at three-month intervals for the first two years and every two treatments thereafter. CIS patients had biopsies 3 or 6 months after start of RF-CHT (this also changed over time, concurrent with guideline recommendations for biopsies in case of CIS evaluation). 

### 4.3. Outcomes

The primary outcome measures were CR and durable response rate or RFS. For patients with (concomitant) CIS or an unresected papillary tumor at baseline, CR at six months was determined which was defined as negative biopsies or negative cystoscopy combined with negative cytology. For (concomitant) CIS patients that reached CR at three or six months, the durable response rate was determined. RFS was determined for papillary patients (without tumor at baseline, thus after a complete TURB, or after CR). An event (recurrence) for durable response and RFS was defined as histology of NMIBC or high-grade cytology. If low-grade recurrence occurred once, the tumor was resected and RF-CHT was continued and this incident was not included as an event in the analysis, analogous to the management of low-grade recurrence during BCG treatment [29]. Patients without recurrence were censored at date of last follow-up or death. 

The secondary outcome measures included progression to MIBC, OS, CSS and RS. Safety and tolerability were assessed in all patients. A registry of the Statistics Netherlands (CBS) was used to monitor if patients were alive [35]. RS is determined as the OS in this cohort divided by the expected survival in a similar Dutch population, based on age, sex and calendar [36]. Moreover, we investigated the association between the treatment duration and the chance of developing a recurrence in patients who completed treatment. The “radical cystectomy free rate”, the proportion of patients in whom a radical cystectomy could be prevented was determined. Adverse events were graded according to CTCAE version 5.0, 2017 [37]. Per symptom, the maximum grade that occurred was included in the database for each patient. Treatment tolerability was evaluated by the drop-out rate because of adverse events. Lastly, the treatment outcome in the BCG refractory subgroup was assessed, defined as patients that have been treated with BCG but discontinued BCG treatment due to refractory or resistant disease, or had relapse during BCG treatment, thus not due to BCG intolerance.

### 4.4. Statistical Analysis

Regarding six-month CR rate, the chi-square test was used to assess the association between the pre-specified factors CIS-status and chemotherapeutic dose. Logistic regression analysis was performed to generate unadjusted and adjusted OR and corresponding *p* values. 

The Kaplan–Meier method was used to generate estimates of RFS, durable response rate, OS, CSS and radical cystectomy -free rate; and to generate a curve for RFS (ablative vs. adjuvant dose). Regarding RFS, subgroups were compared using stratified log-rank test. We used Cox proportional hazard regression to adjust for potential confounders and to give adjusted and unadjusted hazard ratios (HR) and corresponding *p* values.

For both logistic regression (CR) and Cox regression (RFS) analysis, the confounders CIS status, prior BCG treatment, previous tumor grade, previous recurrence rate, chemotherapeutic drug (combined with RF-CHT) were included, in addition to dose (ablative vs. adjuvant, primary comparison). 

The association between treatment duration and the chance of developing a recurrence after end of treatment was also determined using the chi-square test. A two-sided *p* value of 0.05 was considered statistically significant. IBM SPSS Statistics for Windows, version 25.0 (Armonk, NY, USA), was used for all statistical calculations. 

## 5. Conclusions

These results show that RF-CHT is effective and safe in intermediate and high-risk NMIBC patients for whom other intravesical treatments have failed. RF-CHT should be considered in patients who are unwilling or unfit to undergo radical cystectomy. Ablative dose increased RFS and caused a trend towards improved CR, thus should be used in CIS patients or patients with residual papillary tumor at baseline. Additionally, the treatment of patients with RF-CHT for at least two years is recommended. 

## Figures and Tables

**Table 1:** Baseline characteristics of the study population.

Description	Subgroup	Title	≥6 Sessions	All Patients
Total, *n*			274	299
Sex, *n* (%)	Female		60 (21.9)	65 (21.7)
	Male		214 (78.1)	234 (87.3)
Age, median (IQR)			66 (60–74)	67 (60–74)
Baseline histology, *n* (%)	CIS		128 (46.7)	146 (48.8)
		CIS + Ta	49 (17.9)	57 (19.1)
		CIS + T1	23 (8.4)	24 (8.0)
		CIS only	56 (20.4)	65 (21.7)
	Papillary		146 (53.3)	157 (52.5)
		Ta LG ^1^	23 (8.4)	23 (7.7)
		Ta HG ^2^	105 (38.3)	114 (38.1)
		T1 HG	18 (6.6)	20 (6.6)
Recurrence frequency, *n* (%)	≥1/year		188 (69.1)	202 (68.2)
	<1/year		84 (30.9)	94 (31.8)
Risk stratification (EAU), *n* (%)	Intermediate		77 (28.1)	81 (27.1)
	High		197 (71.9)	218 (72.9)
Previous BCG treatment, *n* (%)	Yes		234 (85.4)	255 (85.3)
		Refractory	178 (65.0)	194 (64.9)
		Intolerant	21 (7.7)	23 (7.7)
	No		35 (12.8)	39 (13.0)
Chemotherapeutic agent, *n* (%)	MMC		239 (87.2)	262 (87.6)
	Epirubicin		35 (12.8)	37 (12.4)
Dose, *n* (%)	Ablative		120 (43.8)	133 (44.5)
	Adjuvant		154 (56.2)	166 (55.5)

^1^ LG, low grade; ^2^ HG, high grade.

**Table 2:** Complete response rates.

Variable	Subgroup	% 6-Month CR ^3^	Unadjusted OR ^4^ (95% CI)	*p* Value	Adjusted OR (95% CI)	*p* Value
Overall (*n* = 137)	CIS and papillary patients with tumor at baseline	55.5				
Baseline histology	(Concomitant) CIS (*n* = 116)	56.0	0.86 (0.34–2.19)	0.76	0.35 (0.10–1.21)	0.10
	Papillary (*n* = 21)	52.4				
Dose	Adjuvant (*n* = 46)	45.7	0.55 (0.27–1.12)	0.10	0.49 (0.23–1.08)	0.08
	Ablative (*n* = 91)	60.4				

^3^ Six-month complete response (CR) for CIS patients and patients with papillary tumor at baseline. ^4^ Unadjusted and adjusted odds ratios (OR) were estimated with logistic regression.

**Table 3:** Recurrence free survival and durable response rates.

Variable	Subgroup	% 1-Year RFS ^5^ (95% CI)	% 2-Year RFS (95% CI)	% 5-Year RFS (95% CI)
Overall (*n* = 204)		78.6 (72.9–84.3)	60.3 (53.2–67.4)	38.1 (30.5–45.7)
Baseline histology	(concomitant) CIS (*n* = 70)	79.7 (69.7–89.7)	66.5 (54.3–78.7)	40.3 (25.2–55.4)
	Papillary (*n* = 134)	77.9 (70.8–85.0)	57.5 (48.9–66.1)	37.2 (28.4–46.0)
Dose	Ablative (*n* = 73)	86.9 (78.9–94.9)	71.9 (60.7–83.1)	47.6 (33.3–61.9)
	Adjuvant (*n* = 131)	74.0 (66.4–81.6)	54.2 (45.4–63.0)	33.9 (25.1–42.7)

^5^ RFS estimates with 95% confidence interval (CI) were obtained with Kaplan-Meier method.

**Table 4:** Multivariable analysis of recurrence free survival and durable response.

Variables	Unadjusted HR ^6^ (95% CI)	*p* Value	Adjusted HR (95% CI)	*p* Value
Ablative vs. adjuvant dose	0.64 (0.42–0.98)	0.04	0.54 (0.34–0.85)	0.01
MMC vs. epirubicin	1.12 (0.65–1.93)	0.69	1.23 (0.71–2.14)	0.46
(Concomitant) CIS vs. papillary	0.85 (0.56–1.30)	0.46	0.94 (0.57–1.55)	0.81
Previous BCG vs. BCG naïve	1.91 (1.04–3.50)	0.04	2.07 (1.05–4.08)	0.04
Previous high vs. low tumor grade	1.15 (0.76–1.74)	0.51	1.25 (0.79–1.98)	0.34
Previous recurrence rate ≥1 vs. <1/year	1.64 (1.06–2.52)	0.03	1.39 (0.85–2.28)	0.19

^6^ Adjusted and unadjusted hazard ratios (HR) with 95% confidence interval (CI) per included confounder, estimated with Cox proportional hazard regression (*n* = 204).

**Table 5:** Univariable analysis of overall survival, relative survival, and cancer specific survival.

Survival (*n* = 274)	5-Year, % (95% CI)	10-Year, % (95% CI)
OS ^7^	72.3 (66.4–87.2)	51.0 (43.4–58.6)
RS ^8^	80.6 (74.0–87.1)	65.1 (55.2–75.1)
CSS ^7^	86.6 (81.7–91.5)	77.6 (70.3–84.9)

^7^ Overall survival (OS) and cancer specific survival (CSS) estimates with 95% confidence interval (CI) were obtained with Kaplan-Meier method. ^8^ Relative survival (RS) is determined as the overall survival in this cohort divided by the expected survival in a similar Dutch population.

**Table 6:** Adverse events.

Adverse Event (*n* = 294)	Any Grade ^9^, *n* (%)	Grade 1, *n* (%)	Grade 2, *n* (%)	Grade 3, *n* (%)
Spasms	183 (62.2)	85 (28.9)	93 (31.6)	5 (1.7)
Pain	82 (27.1)	60 (20.1)	17 (5.7)	5 (1.7)
Catheter problems	52 (17.7)	30 (10.2)	18 (6.1)	4 (1.4)
Dysuria	156 (53.1)	126 (42.9)	26 (8.8)	4 (1.4)
Hematuria	88 (29.9)	83 (28.2)	5 (1.7)	0 (0)
Urinary tract infection	46 (15.6)	0 (0)	39 (13.3)	7 (2.4)
Nocturia	43 (14.6)	22 (7.5)	16 (5.4)	5 (1.7)
Incontinence	18 (6.1)	12 (4.1)	6 (2.0)	0 (0)

^9^ Maximum CTCAE grade reported.

## Data Availability

The data presented in this study are available on request from the corresponding author. The data are not publicly available due to privacy issues.

## Appendix A

The appendix includes more specific data for the 274 patients included in the efficacy analysis. Follow-up data of 249 (91%), 212 (77%), 129 (47%) and 59 (22%) patients was available for at least one, two, five and ten years, respectively. Of the total, 111 patients had a recurrence during a median follow-up of 55.5 months. Also, 105 patients died in the period from start of RF-CHT to the moment of retrieving data from the registry of the Statistics Netherlands (CBS; December 2019), a median time 91.5 months.
